# An Overview of the *Alternaria* Genus: Ecology, Pathogenicity and Importance for Agriculture and Human Health

**DOI:** 10.3390/jof12010064

**Published:** 2026-01-13

**Authors:** Stanislava A. Vinogradova, Konstantin V. Kiselev, Andrey R. Suprun

**Affiliations:** 1 Federal Scientific Center of the East Asia Terrestrial Biodiversity, Far Eastern Branch of the Russian Academy of Sciences, 690022 Vladivostok, Russia; vinogradova.sal@dvfu.ru (S.A.V.); kiselev@biosoil.ru (K.V.K.); 2 Department of Medical Biology and Biotechnology, Molecular Biotechnology, Far Eastern Federal University, 690090 Vladivostok, Russia

**Keywords:** *Alternaria*, taxonomy, phytopathogen, alternariosis, mycotoxins, biological control, resistance, biotechnological potential, fungicide resistance

## Abstract

*Alternaria* is a widespread genus and a diverse taxonomic group of fungi, whose members exhibit a wide range of ecological roles, from endophytes and saprophytes to potent plant pathogens, and in some cases, to opportunistic pathogens or allergens affecting humans. Their high adaptability to various environmental conditions determines their widespread distribution and resilience. A key feature of the genus *Alternaria* is its substantial species diversity. According to the Species Fungorum database, it currently comprises 792 registered species, which are grouped into 29 sections. It should be noted that this number reflects the current state of taxonomic classification and is subject to ongoing revision. The ecological role of representatives of this genus is particularly relevant in the context of agriculture, as many species are pathogens and causative agents of *Alternaria* leaf spot in important agricultural plants such as tomatoes, potatoes, apples, wheat, and others. This disease causes significant economic losses. At the same time, some strains demonstrate potential for use in biotechnology due to their ability to produce biologically active metabolites. This review examines the taxonomy, morphological characteristics, ecological role, pathogenicity, and control methods of fungi of the genus *Alternaria*, as well as their biotechnological potential.

## 1. Introduction

Microscopic fungi of the genus *Alternaria* represent one of the most widespread, ecologically flexible and economically significant groups of microorganisms in the kingdom Fungi [1]. Being cosmopolitan, these fungi exhibit an incredible diversity of life strategies, acting as saprotrophs, endophytes, and opportunistic human pathogens [2]. Their role as plant pathogens is highly context-dependent, varying from latent endophytism to aggressive parasitism, which is determined by the specific host species, environmental conditions, and fungal genotype [3]. The unique ability to colonize a wide range of substrates from plant tissues and agricultural products to soil and household dust, makes them the object of close attention from researchers in a wide range of scientific fields: from phytopathology and biotechnology to medicine and ecology [3,4].

The history of the study of the genus *Alternaria* began in 1817 with the description of the species *A. tenuis* by Christian Gottfried Daniel Nees von Esenbeck [5]. Since then, the taxonomy of this group has remained complex and largely controversial. Although almost 800 species are registered in databases, only about 360 are officially recognized based on robust, multi-criteria taxonomic studies, which speaks volumes about the problem of blurred interspecific boundaries [6]. The traditional division into small- and large-spore species, as well as the allocation of 29 sections within the genus, often do not coincide with the conclusions of molecular genetic studies [7]. For instance, the conventional division into small- and large-spore groups, as well as characteristics like colony color and texture, provide useful preliminary data for separating isolates into broad sections (e.g., Porri or Infectoriae) [8,9]. However, these traits are insufficient for accurate delineation at the species or even sectional level, as phylogenetic analyses frequently regroup species, revealing that some morphologically defined sections (such as the broad section Alternata) are polyphyletic [10]. High morphological plasticity, dependence of characteristics on environmental conditions and the presence of strains with intermediate characteristics have long complicated precise identification [11]. Modern taxonomy increasingly relies on DNA barcoding methods and multilocus phylogenetic analysis, but even these do not always allow for the reliable separation of closely related species such as *A. alternata*, *A. arborescens*, and *A. tenuissima* [7,9,12,13]. A breakthrough in this field has been the use of genome-wide analysis studies (GWAS), which recent studies show is the most promising tool for resolving taxonomic controversies [14].

From an ecological perspective, *Alternaria* is a master of adaptation. Many species are capable of an endophytic lifestyle, living inside their host plants and even benefiting them, for example, by increasing their resistance to abiotic stress [2]. However, when the plant is weakened or environmental conditions change, same species easily switch to a parasitic lifestyle [9]. The life cycle is asexual, optimized for rapid dispersal: characteristic dark-colored conidia, forming chains [15]. Conidia are easily spread by wind and raindrops, ensuring population stability in agrocenoses and natural ecosystems [16]. *Alternaria* cause the greatest damage as phytopathogens, causing diseases such as *Alternaria* leaf spot and fruit rot in tomatoes, potatoes, cabbage, cereals and many other crops [17,18,19,20,21,22,23]. Under favorable weather conditions, economic losses from their activities can reach 60–70% of the harvest [24]. Pathogenicity is ensured by a complex arsenal of enzymes and, most importantly, specific secondary metabolites—phytotoxins (AAL, ACR, AF, AM toxins, etc.)—that disrupt the metabolism of host plant cells, leading to tissue necrosis [25,26].

Combating *Alternaria* leaf spot requires a comprehensive approach, including agronomic measures, chemical, biological, and genetic selection methods. However, excessive use of fungicides leads to environmental pollution and the emergence of resistant strains, necessitating the search for alternative solutions, such as the use of antagonistic microorganisms, the development of resistant varieties, and the use of modern biotechnology, including CRISPR/Cas9, though their application remains largely at the research stage [9,27,28].

Paradoxically, while being a source of problems, *Alternaria* fungi also possess significant biotechnological potential. They produce over 300 different metabolites, some of which exhibit herbicidal, antibiotic, and cytotoxic activity, opening up prospects for the development of new drugs, including anticancer ones [29,30,31]. Moreover, it is interesting that the impact of *Alternaria* on human health is not limited to food loss. The spores of these fungi are among the most potent respiratory allergens, and the mycotoxins they produce, such as alternarol (AOH) and its derivatives, pose a serious risk when contaminated food is consumed, demonstrating genotoxic and carcinogenic properties [32,33].

Thus, the genus *Alternaria* represents a complex and multifaceted subject of research, acting simultaneously as a dangerous pathogen, a promising source of biologically active compounds, and a significant ecological factor. In contrast to publications focused on individual aspects of *Alternaria* biology (taxonomy, mycotoxins, control methods), this review provides a comprehensive analysis that integrates fundamental and applied areas of research on the genus. Primary focus is given to the modern taxonomic system, a detailed analysis of the molecular mechanisms of pathogenicity including the subcellular targets of host-specific toxins, as well as a critical review of both traditional and prospective control strategies, including biotechnological approaches. The work systematizes current data and offers a holistic view of the interrelationship between ecology, pathogenesis, and the practical significance of *Alternaria*. The purpose of the review is to consolidate current knowledge on the taxonomy, ecology, pathogenicity, control methods, and practical potential of these fungi, which is an important task for developing strategies for sustainable agriculture, biotechnology, and human health protection.

## 2. Taxonomy

In 1817, Christian Gottfried Daniel Nees von Esenbeck first identified the genus *Alternaria* and described *A. tenuis* as its species [5]. Currently, the genus *Alternaria* belongs to the family Pleosporaceae (Pleosporales, Dothideomycetes, Ascomycota) and has 792 species registered in the Species Fungorum database (https://www.speciesfungorum.org, accessed on 26 May 2025), but due to the unresolved taxonomy of the genus, only 360 species are officially recognized [6]. This genus includes two conventional groups—small-spore and large-spore species, which, based on morphological and molecular genetic approaches, form several sections that do not always correlate with species groups identified on the basis of morphological characteristics [7,11]. It is important to note that in some cases, it is difficult to draw a clear line between small-spore and large-spore *Alternaria* species due to the lack of clear criteria for distinguishing these groups. Currently, 29 sections have been identified within the genus *Alternaria*: Alternantherae, *Alternaria*, Brassisicola, Chalastospora, Cheiranthus, Crivellia, Dianthicola, Embellisia, Embellisioides, Euphorbiicola, Eureka, Gypsophilae, Infectoriae, Japonicae, Nimbya, Panax, Phragmosporae, Porri, Pseudoalternaria, Pseudoulocladium, Radicina, Soda, Sonchi, Teretispora, Ulocladioides, Ulocladium, Undifilum [1,7,9]. Morphological characters have long been a universal tool for classifying strains of *Alternaria* and related genera [34]. However, this approach is not always effective due to the high influence of growing conditions on morphological characteristics, the high level of similarity between some species and the presence of several strains with intermediate characteristics [35]. High morphological plasticity within the genus *Alternaria* also complicates identification based solely on morphological characteristics. DNA barcoding technology is currently the most widely used method in the field of molecular species identification [6,9,12]. For the species identification of *Alternaria*, a combination of molecular markers is employed. The core markers, essential for robust phylogenetic placement, typically include the internal transcribed spacer (*ITS*) region, glyceraldehyde-3-phosphate dehydrogenase (*GAPDH*), RNA polymerase second largest subunit (*RPB2*), and the translation elongation factor 1-alpha (*TEF1*) [1,9,29]. Auxiliary markers, such as the *Alternaria* major allergen (*Alt a 1*), endopolygalacturonase (EndoPG) gene, the anonymous region OPA10-2, and the ribosomal genes 18S nrDNA (SSU) and 28S nrDNA (LSU), provide additional resolution and are used to clarify relationships within specific species-groups or complexes [30].

For the species identification of *Alternaria*, the most commonly used gene markers are internal transcribed spacer (*ITS*), glyceraldehyde-3-phosphate dehydrogenase (*GADPH*), RNA polymerase second largest subunit (*RPB2*), *Alternaria* major allergen (*Alt-a1*), endopolygalacturonase (*EndoPG*) gene and an anonymous gene region (*OPA10-2*), translation elongation factor 1 alpha (*TEF1*), 18S nrDNA (*SSU*), 28S nrDNA (*LSU*) [1,13,35,36]. Typically, species identification is based on the analysis of the nucleotide sequence of several gene markers, usually at least three. However, the genetic sequences of the loci commonly used for species identification are poorly differentiated in small-spore species, making their distinction problematic [37]. In particular, *ITS*, which is the standard genetic barcode for identifying fungi, is often not informative enough to distinguish closely related species [38]. For example, the *ITS* sequences of *A. brassicae*, *A. alternata*, *A. porri*, *A. infectoria*, and *A. tenuissima* have a high degree of homology, making it impossible to reliably differentiate them based on the *ITS* region alone [39]. Moreover, even multilocus phylogenetic analysis does not always allow for the reliable separation of morphologically similar species [35]. It is worth mentioning that species identification can also be based on specific secondary metabolites [40]. While secondary metabolite profiles are a valuable diagnostic tool and can be used for the identification of some species, their resolving power is often insufficient for reliably distinguishing morphologically similar and phylogenetically close species, such as *A. al-ternata*, *A. arborescens*, and *A. tenuissima* [17,40]. This limitation stems from the fact that the synthesis of many secondary metabolites in fungi of the genus *Alternaria* is conserved within species complexes or can vary depending on environmental conditions and developmental stage, which does not always reflect true phylogenetic boundaries [40]. Therefore, similar to multilocus phylogenetic analysis, chemotaxonomy in this context cannot serve as the sole criterion for species identification.

The insufficient taxonomic knowledge of *Alternaria*, manifested in blurred interspecific boundaries, represents another key problem. The debate continues between those who advocate for merging similar species and researchers who argue for the possibility of distinguishing them using multigene analysis [35,38,41]. Currently, the most promising and reliable method of species identification is the use of the whole genome analysis (GWAS) method [14]. The advantage of GWAS is that whole genome sequences of different species inevitably exhibit differences [6,14]. However, it is not yet a routine identification tool, as its reliability depends on high-quality, well-annotated reference genomes, and its use is currently limited by cost, technical accessibility, and computational demands. Yang et al. (2025) successfully used the AGE method to identify seven *Alternaria* species—*A. arbusti*, *A. infectoria*, *A. solani*, *A. tenuissima*, *A. triticina*, *A. alternata* and *A. longipes* [6].

## 3. Ecology and Distribution

*Alternaria* is a ubiquitous genus of fungi characterized by significant species diversity, including endophytic and saprophytic species, as well as plant and human pathogens [2]. A key feature of these fungi is their high degree of adaptability and fitness, which enables them to successfully colonize and inhabit diverse ecosystems and under changing environmental conditions. This allows them to colonize a wide range of substrates: from seeds and vegetative parts of plants to agricultural products, as well as to infect animals and persist in soil [9,32,42,43].

Representatives of this genus exhibit exceptional life cycle variability, allowing them to adapt to a wide variety of environmental conditions. Many species can exist as endophytes, living inside plants without causing visible disease symptoms [2,9]. There is evidence that certain strains of endophytic *Alternaria* species may have a positive effect on plant growth and development, while others may be neutral or pathogenic [44,45,46]. This effect is often associated with the production of various secondary metabolites by the fungus, which may play a role not only in the adaptation of the fungus itself, but also, probably, contribute to the stimulation of plant growth, increasing its resistance to abiotic stresses (drought or salinity) or enhancing protection against other pests [42,47]. *A. alternata* produces ergosterol, β-sitosterol, ergosterol peroxide, fonsecinone A, asperopyrone C, and asperopyrone B. These compounds are able to significantly inhibit the growth of bacteria such as *Pseudomonas aeruginosa*, *Salmonella enterica*, *Escherichia coli* and *Klebsiella pneumoniae* [48]. *A. oxytropis* is capable of synthesizing indole-3-acetic acid (auxin) as a secondary metabolite, thereby directly influencing plant growth and development (root system development, increase in root hairs, improved absorption of water and nutrients, etc.) [49]. The fungus is also capable of producing other compounds (CO_2_ or ethylene), influencing plant development by regulating the auxin signaling pathway [49]. In the absence of suitable living hosts (or after their death), the fungus can effectively transition to a saprophytic lifestyle, feeding on dead and decaying organic matter. This “dormant” stage is necessary to maintain the population in the environment, ensuring its viability while awaiting favorable conditions or the emergence of new hosts [9,11,50]. Also, when environmental conditions change or the host plant weakens, both endophytic and saprophytic species of *Alternaria* can switch to a parasitic lifestyle, exhibiting pathogenic properties and causing diseases [51]. This transition is often mediated by changes in fungal gene expression, including the upregulation of virulence factors such as host-specific toxins, cell wall-degrading enzymes, and effector proteins that suppress plant immunity [2,42]. The shift from a balanced interaction to parasitism is thus a consequence of altered molecular dialog between the fungus and its host under stress.

## 4. Morphology and Life Cycle

The main distinguishing feature of fungi of the genus *Alternaria* is the presence of multicellular spores (conidia), which are typically oval or teardrop-shaped, characterized by multiple septations, and a dark brown color. They reproduce predominantly asexually via the formation of conidia, as sexual stages (teleomorphs) have rarely been observed or are unknown [52]. Conidia may form long unbranched chains, chains with lateral branches, or be singly arranged. Conidia range in size from 20 to 50 × 8.75-21 µm. The mycelium is substratum and sometimes superficial; hyphae are colorless, olive, or brown [9]. The colonies are round in shape with a hairy structure, the color varies from light gray to dark brown or black, which is due to the presence of dihydroxynaphthalene melanin (DHN melanin) in the conidia and mycelium [9,11,18]. Under unfavorable conditions, such as low temperature, lack of moisture, high concentration of toxic substances in the environment or nutrient deficiency, chlamydospores can form from the terminal or intercal cells of hyphae, which can subsequently combine to form sclerotia that can safely overwinter in the soil [50,53]. It should be noted that not all *Alternaria* species form such structures.

Optimal conditions for the growth and development of representatives of the genus *Alternaria* show some variability depending on the species. In general, they behave as mesophilic organisms, preferring moderate temperatures, and demonstrate good growth on standard nutrient media such as PDA (Potato Dextrose Agar), CMA (Corn Meal Agar), etc. Summarizing the data on various species, it can be concluded that the most favorable conditions for growth and sporulation are air temperatures in the range of 20–30 °C and high humidity (80–100%), and they prefer neutral or slightly acidic environmental conditions [3,7,9].

Plant infection occurs under conditions of high temperature and humidity. The first symptoms appear within 3–5 days [16,54]. It is generally accepted that fungal infection of plants is divided into three main stages: (I) the initial stage (adhesion and germination), (II) the penetration stage and (III) the colonization stage [55,56]. I: The initial stage occurs between 8 and 32 °C and humidity from 80 to 100% and involves swelling and germination of conidia. II: The penetration stage begins with the formation of appressoria—modifications of the mycelium that allow hyphae to penetrate the host, attaching the fungus to the plant surface. Next, hyphae grow into host cells and branch. Pathogen penetration is also possible through stomata between plant epidermal cells or by enzyme-mediated destruction of the plant cell wall. III: The colonization stage involves further hyphal germination on the surface or within plant tissues. New conidia are formed at each stage: they can form from a germinating conidium (stage I), as well as at the tips of short, unbranched conidiophores (stage III) [7,50,56]. Spore formation typically requires prolonged moisture, but can also occur during alternating wet and dry periods. Conidiophores form during the damp night, and then the next wet night after a dry day produces conidia. These conidia are then quickly dispersed by wind and raindrops, infecting healthy areas of the same plant or neighboring plants [16]. It was also noted that with an increase in the duration of leaf wetting, the severity of the disease increases at all temperatures [54].

## 5. Pathogenicity for Plants

**Figure 1:**
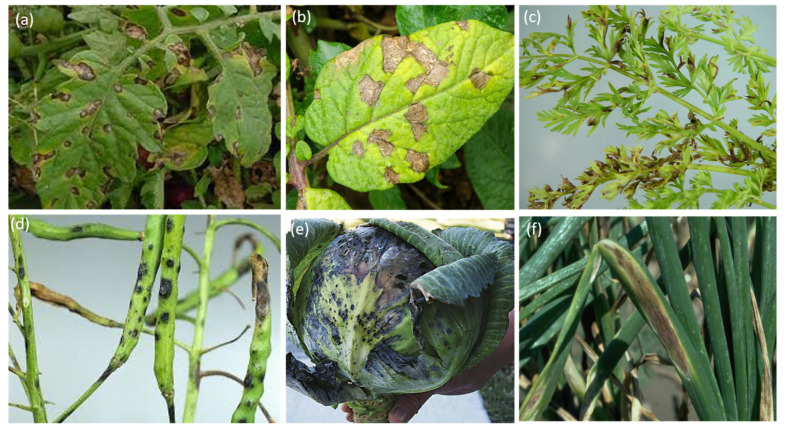
Images of *Alternaria* symptoms on (**a**) tomato, (**b**) potato, (**c**) carrots, (**d**) rapeseed, (**e**) cabbage, and (**f**) onion.

Fungi of the genus *Alternaria* are among the most dangerous phytopathogens, causing significant economic losses in agriculture. Typically, this group of phytopathogens includes species from the sections Porri, Brassicicola, Radicina, and Japonicae [7,57]. They can infect a wide range of crops, including cereals, ornamentals, tomatoes, potatoes, carrots, rapeseed and cabbage, causing diseases such as *Alternaria* leaf and fruit rot, spotting and post-harvest rot (Figure 1) [11].

**Figure 2:**
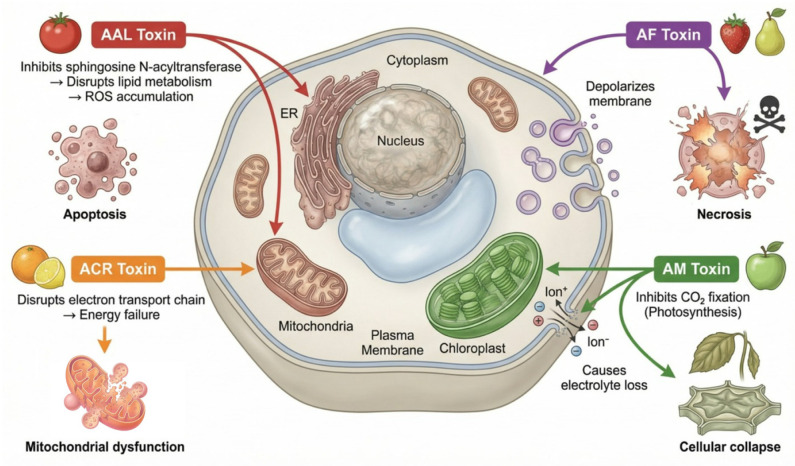
Schematic model of *Alternaria* toxins action on plant cellular targets.

Fungal pathogens, including *Alternaria*, produce a complex of enzymes and secondary metabolites that facilitate plant infection. These compounds can destroy host cellular structures or modify their metabolism [58,59]. Different strains and species, depending on the affected plant, produce specific toxins, such as AAL toxin (produced during tomato damage; affects the endoplasmic reticulum and mitochondria, prevents lipid production by sphingosine N-acyltransferase, thereby causing ROS accumulation and subsequent cell apoptosis), ACR toxin (produced during citrus damage; affects mitochondria, disrupting their function by disrupting the electron transport chain), AF toxin (produced during strawberry and pear damage; depolarizes the plasma membrane, leading to invagination, fragmentation, vesiculation and subsequent cell necrosis), AM toxin (produced during apple damage; affects both chloroplasts and the plasma membrane of leaf cells, inhibiting photosynthetic CO_2_ fixation and causing electrolyte loss), etc. (Figure 2) [60,61,62]. In addition, *Alternaria* spp. produce other phytotoxins such as brefeldin, tentoxin and maculosin destruxin B, which play a role in pathogenesis [11].

Despite morphological and molecular genetic differences between various *Alternaria* species, they cause similar infection patterns [18]. However, their infection patterns diverge significantly in host specificity and the nature of symptoms they induce, ranging from leaf spots and blights on specific plants to post-harvest rots on stored produce. Infection leads to the appearance of necrotic lesions in approximately 5–7 days, with a characteristic yellowing of the senescent tissue, which typically occurs due to the diffusion of fungal phytotoxins. Sporulation at the lesion sites facilitates further spread of the disease, spreading to leaves, stems, fruits, and tubers. Primary foci of infection are often barely noticeable, but subsequent massive spore production (secondary sporulation) leads to severe disease development in the later stages of vegetation [63].

The fungus *A. solani* causes early blight on potatoes and tomatoes, affecting leaves, stems, tubers, and fruits. During severe outbreaks in conducive environments, yield losses due to this disease have been reported to reach up to 60–70% for tomato fruits and up to 20–30% for potato tubers [50,64]. *A. porri* causes early blight of leek, which causes massive losses of seed and bulb yields worldwide [65]. *A. bataticola* infects sweet potato in tropical and subtropical countries, causing losses of more than 70% of the sweet potato crop [35]. Among the most common pathogens are *A. brassicicola*, *A. japonica* and *A. brassicae*, which cause *Alternaria* blight in cruciferous crops (rapeseed, canola, radish, radish, turnip, watercress, garden mustard, etc.) (Table 1) [9,43]. In addition, *A. japonica* is a pathogen of plants of the genus *Raphanus*, causing leaf spot of radish [25]. Several species of *Alternaria* also cause damage to leaves and grains of wheat [26]. *A. triticina* has been shown to be able to infect wheat, causing leaf spot [66]. However, *A. alternata*, *A. arborescens*, *A. infectoria*, *A. tenuissima* and *A. triticina* are also known to be the typical cause of black germ disease of wheat grains, which is a serious problem in global agriculture [26,34,67]. *A. tenuissima* attacks sugar beet, causing leaf spot, which appears as dark brown spots and tissue necrosis [68]. *A. alternata* causes leaf spot in aloe vera by destroying the mesophyll tissue of the leaf and thereby reducing the antimicrobial potential of the gel [69]. *A. alternata* can also affect most types of tea plants, in particular *Camellia sinensis*; the disease appears on young leaves in the form of necrotic spots, reducing the yield and quality of the product [70]. *A. poonensis* and *A. alternata* cause leaf spot on coriander plants and are also seed-borne [71].

## 6. Methods of Control of *Alternaria* in Plants

**Figure 3:**
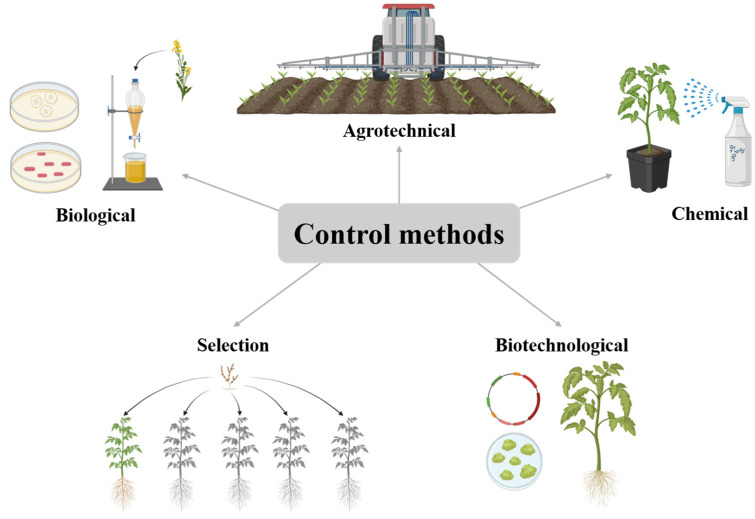
Schematic representation of methods for control of plant diseases caused by *Alternaria* spp.

Methods for controlling phytopathogenic *Alternaria* fungi include a complex of agronomic, chemical, biological, breeding and biotechnological approaches (Figure 3).

### 6.1. Agrotechnical Approach

Agronomic practices include the use of healthy and treated (by dressing or treatment with hot water at a temperature of 55–60 °C for 10–30 min) seeds, maintaining a long crop rotation (3–4 years) with periods of fallowing, the correct density (45 × 30 cm) and depth (~2 cm) of planting crops, maintaining sanitation in the fields, weed control and destruction of crop residues [7,43,87]. Interestingly, ultraviolet-C (UV-C) seed treatment can also be used to control *Alternaria* spp. Hahlbohm et al. (2025) showed that UV-C treatment of spores resulted in reduced spore germination of all fungal species, including *Alternaria* spp. [88]. It is also recommended to avoid overwatering caused by overhead watering by using drip irrigation to reduce leaf wetness and thus the risk of infection [7].

### 6.2. Chemical Approach

Chemical control methods rely on fungicides, which inhibit pathogen growth and prevent disease progression. They can be used both prophylactically and directly to treat already affected plants [89].

Azoles are a group of compounds containing aromatic five-membered rings with nitrogen atoms. They are divided into imidazoles (a ring with two nitrogen atoms) and triazoles (rings with three nitrogen atoms). They inhibit the enzymes lanosterol-14α-demethylase and CYP51, which is necessary for the biosynthesis of ergosterol, a key component of the fungal cell membrane, thereby suppressing fungal growth. Typically, their use is most effective on infected plants in the early stages, before the fungus has formed spores. The most commonly used are propiconazole, tebuconazole, and difenoconazole [90,91]. For example, in the work of Yurchenko et al., it was found that difenoconazole demonstrates high efficiency in suppressing *Alternaria* spp. and is recommended for integrated grape protection schemes [92].

Strobilurins (QoI fungicides) are a group of compounds derived from natural fungicidal toxins isolated from the fungus *Strobilurus tenacellus*. They inhibit cellular respiration by specifically binding to the quinol oxidation site (Qo) on cytochrome b, which leads to the interruption of electron transfer between cytochromes b and c1 and further suppression of nicotinamide adenine dinucleotide (NADH) and adenosine triphosphate (ATP) synthesis [93]. Of contact fungicides with a long action time, the most common are trifloxystrobin and azoxystrobin [94,95]. Wang et al. (2016) showed that azoxystrobin is superior to difenoconazole in controlling conidial germination of *A. alternate* [96], which is also consistent with previously obtained results for the same group of chemicals in other *Alternaria* species [97,98,99,100].

Dithiocarbamates are a group of fungicides derived from dimethyldithiocarbamic and ethylenebisdithiocarbamic acids. When exposed to water, they decompose, releasing ethylene bis-isothiocyanate sulfide (EBIS), which can inhibit biochemical processes in both the cytoplasm and mitochondria of fungi [101]. EBIS inhibits enzymes containing sulfhydryl groups or metal atoms involved in lipid metabolism, cellular respiration, and ATP synthesis. They are non-specific contact fungicides, and thiram and mancozeb are often used [102]. According to a study by Gondal et al., the use of mancozeb in various concentrations (from 4 to 28 g/L) effectively inhibits the growth and development of the pathogen, reducing the severity of the disease [103].

A major advance in the chemical control of pathogens, including *Alternaria* spp., has been the introduction of succinate dehydrogenase inhibitors (SDHIs) [104]. These fungicides target a key enzyme in the mitochondrial respiratory chain, complex II (succinate dehydrogenase), blocking electron transfer and thereby disrupting the fungus’s energy metabolism [105]. Due to their specific mechanism of action, SDHIs demonstrate high efficiency in protecting a wide range of agricultural crops, exhibiting both preventive and therapeutic activity [104]. The most well-known representatives of this class used against *Alternaria* include boscalid, penthiopyrad and fluopyram [106]. However, as with other fungicides, intensive use of SDHI has led to the emergence and spread of resistant pathogen strains, which is a serious problem [107,108]. Therefore, this class is recommended for use within the framework of anti-resistance strategies. For example, in mixtures or alternation with preparations of other chemical groups, which emphasizes the importance of their consideration in the overall plant protection system [106].

Although fungicides are effective, their excessive use can cause numerous environmental problems: substances formed during their breakdown can remain in the soil, leading to contamination, and some fungicides can be toxic to non-target organisms, including humans, insects, etc. Also, with prolonged exposure to certain fungicides, fungi can develop resistance, requiring the use of other products or completely different methods of pathogen control [87,109].

### 6.3. Biological Approach

Biological control is an environmentally friendly method of combating plant pathogens that meets all environmental standards without causing harmful effects on crops and people [28]. Antagonistic microorganisms, such as *Trichoderma*, *Bacillus* and *Pseudomonas*, are typically used as biological agents to control *Alternaria* [110,111]. Microorganisms can effectively inhibit fungal growth by preventing mycelial growth and spore formation. They are used in a variety of ways, including seed treatment, soil application, or foliar spraying [87]. The effectiveness of plant infection control is increased by the combined use of biological (e.g., strains such as *T. viride*, *T. afroharzianum*, *P. fluorescens*, *Penicillium chrysogenum*, *B. subtilis*, *Streptomyces hygroscopicus*, etc.) and chemical fungicides [89]. Various plant extracts are also used, for example, from the cloves of *Allium sativum* (has inhibitory potential against mycelial growth and sporulation in vitro), from neem leaves (suppress mycelial growth), from the leaves and stems of *Myoporum bontioides*, etc. [71,87].

### 6.4. Selective Approach

The most effective and long-term approach to combating *Alternaria* is the development and/or selection of plants with natural resistance to fungal infections, which is achieved through physical barriers, biochemical and immune responses, and the production of secondary metabolites. By using resistant varieties, the use of chemical control methods can be significantly reduced, thereby helping to avoid environmental pollution and the development of resistance in pathogens [7]. Plant physical barriers are the first line of defense against pathogens, including *Alternaria*. These include the cuticle—a waxy layer on leaves and stems that can prevent pathogen penetration; stomata, which also prevent entry when closed; and various bristles, which typically hinder movement within the plant [112].

Plant secondary metabolites also play an important role in plant defense against various pathogens [113]. Phenolic compounds inhibit the growth and development of microorganisms such as fungi and bacteria, directly killing or inhibiting their reproduction [114]. Furthermore, lignin (a polymer of phenolic acids) is found in plant cells and can increase the strength and rigidity of the cell wall, thereby making it less permeable to pathogens [115]. They also stimulate the production of other substances and enzymes, such as phenylalanine ammonia lyase, which cleaves ammonia from phenylalanine to form trans-cinnamic acid, a precursor to various phenolic compounds—a positive feedback pathway [116]. It has been found that when plants are exposed to any type of stress (biotic or abiotic), the levels of phenolic compounds in plants increase [117,118].

The presence of alkaloids also improves plant resistance to various stresses. These compounds exhibit antimicrobial and antioxidant activity, which helps suppress pathogen growth and strengthen plant immunity [119]. Putrescine is a polyamine alkaloid primarily involved in plant defense through oxidation, producing ROS, which in turn induce oxidative stress and subsequently apoptosis of pathogen cells [120,121]. Studies show that resistant plant varieties have significantly higher levels of phenolic compounds and alkaloids compared to susceptible varieties. For example, the resistant tomato variety V19 showed a 104.7% increase in phenolic compounds and a 100% increase in alkaloids and terpenoids [113].

### 6.5. Biotechnological Approach

Genome editing technologies (CRISPR/Cas9) and RNA interference (RNAi) are also effectively used to combat *Alternaria*, aimed at suppressing genes responsible for toxin biosynthesis, pathogenicity and host specificity, which are included in conditionally dispensable chromosomes (CDC), presumably acquired through horizontal transfer from other pathogens [109].

For example, genes encoding polyketide synthases (*Pks*)—enzymes that play a key role in the initial stages of the biosynthesis of many fungal secondary metabolites, including alternariol, AAL toxin, ACR toxin, AF toxin, AM toxin, etc. [60,62]. According to a study by Johnson et al., genes involved in toxin synthesis, including *Pks*, may be located on a conditionally dispensable (CD) chromosome, the loss of which results in the fungus losing its ability to produce toxins and thus ceasing its plant pathogenicity [122].

However, another, complementary approach involves genetically modifying host plants to enhance their innate resistance not only to *Alternaria* but also to other pathogens [123]. For example, overexpression of pathogenesis-related (PR) proteins leads to increased resistance to pathogenic fungi in some crops. In particular, the enzyme chitinase, which is capable of degrading the cell walls of invading phytopathogenic fungi, plays an important role in plant defense responses [124]. Transgenic plants in which the chitinase gene was placed under the control of the overexpressing CaMV 35S promoter were shown to suppress *A. brassicae* colony size by 12–56%, reduce the number of lesions, and delay disease onset compared to non-transgenic controls [125].

It is now well known that when attacked by pathogens, plants increase their synthesis of jasmonate (JA), which helps suppress necrotrophic pathogens that feed on plant cells [126]. For example, in response to *A. alternata* invasion, wild tobacco not only activates the ABA signaling pathway, which leads to stomatal closure, but also engages the ethylene and JA signaling systems, which regulate scopoletin biosynthesis and increase plant resistance [127]. In Arabidopsis thaliana, protection against *Alternaria* pathogens is mediated by the synergistic interaction of JA, salicylic acid (SA), and abscisic acid (ABA) [128]. In the case of chrysanthemum, transcriptome analysis identified a candidate gene that positively regulates SA biosynthesis, the overexpression of which in susceptible lines enhanced their resistance to the necrotrophic fungus *Alternaria* sp. [129].

Various families of transcription factors, such as AP2/ERF, bHLH, bZIP, MYB, NAC, and WRKY, play key roles in the response to pathogens [130]. Recent studies have shown that the transcription factor NaWRKY70 increases resistance to A. alternata by controlling the biosynthesis of JA-Ile, ABA, and capsidiol [131]. In addition, the complex plant defense response includes the generation of reactive oxygen species (ROS), hypersensitive responses (HR), stomatal closure, and cell wall remodeling [132].

## 7. Biotechnological Potential of *Alternaria* Fungi

Although the genus *Alternaria* is primarily known as a plant pathogen and producer of mycotoxins and allergens, it also possesses biotechnological potential, primarily due to its ability to synthesize various secondary metabolites. Over the past few decades, more than 300 metabolites of *Alternaria* fungi have been discovered, some of which exhibit phytotoxic, antibiotic, antifungal, and antiprotozoal activity [133,134]. Currently, all metabolites of fungi of the genus *Alternaria* can be divided into several groups: nitrogen-containing compounds, steroids, terpenoids, pyranones (pyrones), quinones, phenols, etc. (Table 2) [135].

Some secondary metabolites exhibit pronounced phytotoxicity, causing harm to plants, and have significant potential for use as herbicides [58]. For example, α, β-dehydrocurvularin and brefeldin A, obtained from *A. zinnia*, exhibit phytotoxic activity against *Xanthium occidentale*, the most harmful weed of summer crops in Australia [136]. Alternethanoxin A and B are polycyclic ethanones from *A. sonchi* that are capable of suppressing the growth of *Sonchus arvensis*, a widespread crop weed that is one of the most difficult to control worldwide [137].

Maculosin, produced by *A. alternata*, exhibits activity against a wide range of plants, such as *Datura stramonium*, *Lycopersicon esculentum*, *Galium aparine*, *Lantana camara* and others. α-acetylorcinol, isolated from *A. dauci*, is active against plants such as tobacco (*Nicotiana alata*), marigold (*Tagetes erecta*), and parsley (*Petroselinum crispum*).

**Table 2:** Phytotoxins of *Alternaria* species against various species.

Phytotoxin Name	*Alternaria* Species	Target Species	Reference
AAL-toxin	*A. alternata*	*Datura stramonium*	[138]
		*Lycopersicon esculentum*	[139]
AAC-toxin		*Ageratina adenophora*	[140]
Tenuazonic acid		*Lantana camara*	[141]
Maculosin		*Centaurea maculosa*	[142]
Tentoxin		*Galium aparine*	[143]
Isotentoxin			
Alternariol 9-Methyl Ether		Spinach	[144]
Alteichin	*A. eichorniae*	*Eichhornia crassipes*	[145]
Alternetanoxin A	*A. sonchi*	*Sonchus arvensis*	[137]
Alternetanoxin B			[137]
Alternetanoxin C			[146]
Alternetanoxin D			[146]
Alternetanoxin E			[146]
Brefeldin A	*A. zinniae*	*Xanthium occidentale*	[136]
Alternariol	*Alternaria* sp.	*Xanthium italicum*; *Pennisetum alopecuroides*	[31]
Altenuisol			
Aldaulactone	*A. dauci*	*Daucus carota*	[59]
Zinniol			[147]
*α*-acetylorcinol		*Nicotiana alata*; *Tagetes erecta*; *Pastinaca sativa*; *Petroselinum crispum*	[58]
*P*-hydroxybenzoic acid			
Culture filtrate	*A. macrospora*	*Parthenium hysterophorus*	[148]
Brassicicolin A	*A. brassicicola*	*Brassica juncea*	[149]
Culture filtrate	*A. tagetica*	*Tagetes erecta*	[150]
Destruxin B	*A. brassicae*	*Salix alba*	[151]
Homozinniol	*A. solani*	*Solanum tuberosum*; *Lycopersicon esculentum*	[152]
Culture filtrate	*A. alternantherae*	*Alternanthera philoxeroides*	[153]

## 8. The Impact of *Alternaria* Fungi on Human Health

Despite their widespread distribution in nature, members of the genus *Alternaria* are opportunistic infectious agents, causing disease primarily in immunocompromised individuals, including patients with immunodeficiency conditions, cancer, or those receiving immunosuppressive therapy [9,18]. Diseases caused by *Alternaria* in humans typically involve skin or subcutaneous infections, but onychomycosis (nail infections), eye infections, fungal rhinosinusitis, osteomyelitis (bone infections), cerebral mycosis, and disseminated mycosis have also been observed. Infection occurs primarily through inhalation of spores or their introduction into the bloodstream through trauma [154]. Maximum airborne spore concentrations are typically observed in regions with warm climates; in temperate regions, peak spore concentrations occur in late summer and fall. Introduced via outdoor air, spores are also widely distributed indoors, where they are often found in household dust and textiles such as carpets and bedding, regardless of the season [29,30]. In general, human cases of infectious *Alternaria* respond well to treatment with well-known antifungal drugs such as fluconazole, miconazole, etc. [18].

In addition to their ability to cause infectious diseases, members of the *Alternaria* genus are also well known for their allergenic potential [154,155]. Some species, mainly small-spore bacteria, can produce proteins that can cause respiratory diseases such as allergies, asthma and pneumonitis [156,157]. The minimum concentration of spores in the air that causes allergic reactions is significantly lower than that of many other mycoallergens (100 spores per m^3^) [7,18]. At least 17 allergenic proteins produced by saprotrophic strains of *A. alternata* are currently known. One of the main allergens is the Alt a1 protein [30,158]. Ramires et al. (2018) [26] showed that of 54 *Alternaria* strains analyzed for mycotoxins, all strains produced alternariol (AOH), alternariol-monomethyl ether (AME), 40 strains produced tenuazonic acid (TA), and 26 strains (63%) produced altenuene (ALT). This study indicates a high potential risk of mycotoxin poisoning from consuming wheat contaminated with *Alternaria* [26]. Consumption of wheat heavily contaminated with AOH and AME has been shown to increase the incidence of esophageal cancer in humans [159]. A study by Solhaug et al. (2015) showed that AOH and AME are genotoxic, mutagenic and can cause DNA double-strand breaks with subsequent cell cycle arrest [33].

Some secondary metabolites with cytotoxic properties are considered as potential agents for cancer chemoprevention. Alternariol, the most common metabolite of *Alternaria* fungi, exhibits cytotoxic activity against L5178Y mouse lymphoma cells [31,160] and can also cause cell death by activating the mitochondrial apoptotic pathway in HCT116 human colon carcinoma cells with an IC50 value of 65 μM [161]. It has also been shown that the fungus *Alternaria sonchi* S-102 produces the antibiotic chloromonilicin and intermediate products [162].

## 9. Conclusions and Prospects

Analysis of contemporary scientific data allows us to conclude that fungi of the genus *Alternaria* represent a global and multifaceted problem with serious implications for agriculture, the food industry, and human health. Despite a long history of research, this genus remains a focus of active investigation, driven by its exceptional ecological plasticity, taxonomic complexity, and the significant economic damage it causes.

A key challenge in the study of *Alternaria* remains the unresolved taxonomic issue. Blurred morphological boundaries between species and the insufficient resolution of standard molecular markers complicate accurate identification, which is critical for phytosanitary control and diagnostics. In this regard, the most promising approach appears to be the implementation of whole-genome analysis (WGA) methods, which can reveal fundamental genetic differences and establish clear species boundaries. Further work in systematics will form the basis for more effective monitoring and forecasting of the spread of pathogenic species.

The epidemiological significance of alternarioses dictates the need to improve comprehensive plant protection systems. As the review shows, no single method is a panacea. Chemical fungicides, while remaining a powerful tool, face the problems of resistance and environmental risks. Consequently, future strategies should focus on an integrated approach, combining the breeding of resistant cultivars based on a deep understanding of plant immunity, the application of biological agents, and the development of eco-friendly, targeted fungicides. Particular hopes are placed on biotechnological methods, such as CRISPR/Cas9 and RNA interference, which allow for precise targeting of both the pathogen’s virulence genes and the host plant’s resistance genes.

Paradoxically, while being a source of threats, fungi of the genus *Alternaria* simultaneously offer significant prospects for biotechnology. Their ability to produce a wide range of biologically active secondary metabolites—phytotoxins, antibiotics, cytostatics—points to their potential for creating new herbicides, antimicrobial, and antitumor drugs. Thus, the study of the metabolomic diversity of *Alternaria* is another promising direction, transforming the pathogen into a valuable biological resource.

An equally important aspect is the impact of *Alternaria* on human health. The allergenic potential of spores and the danger of mycotoxicoses associated with the consumption of contaminated products require enhanced quality control of grains, fruits, and vegetables at all stages of the production chain—from field to shelf. The development of sensitive detection methods for major allergens (such as *Alt a 1*) and mycotoxins (e.g., AOH, AME) is a prerequisite for ensuring food safety.

In conclusion, the genus *Alternaria* can be viewed as a model for studying evolutionary adaptation, ecological plasticity, and pathogenesis in fungi. Further interdisciplinary research, uniting the efforts of mycologists, phytopathologists, geneticists, biotechnologists, and medical scientists, will not only help mitigate the negative impact of these organisms but also unlock their unique potential for the benefit of humanity.

## Figures and Tables

**Table 1:** Host range of *Alternaria* species.

*Alternaria* Species	Host	Affected Area	Reference
*A. solani*	Potato (*Solanum tuberosum*)	Leaves, stems, tubers and fruits	[50,64]
	Tomato (*Solanum lycopersicum*)		
*A. porri*	Onion (*Allium ampeloprasum*)	Leaves, seeds and bulbs	[65]
*A. bataticola*	Sweet potato (*Ipomoea batatas*)	Leaf and Stem	[35]
*A. brassicicola*	Broccoli (*Brassica oleracea* var. italica)	Leaves	[56,72]
	White cabbage (*Brassica oleracea* var. capitata f. alba)	Leaves	[73]
	Arabidopsis (*Arabidopsis thaliana*)	Leaves	[19]
*A. brassicae*	Canola (*Brassica napus*)	Leaves	[74]
	Mustard (*Brassica juncea*)	Leaves	
	White cabbage (*Brassica oleracea* var. capitata f. alba)	Leaves	[56]
*A. japonica*	*Orychophragmus violaceus*	Leaves	[20]
	Cultivated rocket (*Eruca vesicaria*)	Leaves	[25]
	Wild rocket (*Diplotaxis tenuifolia*)		
	Rape (*Brassica napus*)	Leaves	[74]
	Mustard (*Brassica juncea*)	Leaves	
*A. triticina*	Wheat (*Triticum aestivum*)	Leaves, grains	[26,66]
*A. alternata*	Wheat (*Triticum aestivum*)	Grains	[26]
	Aloe vera (*Aloë vera*)	Leaves	[69]
	Tea (*Camellia sinensis*)	Leaves	[70]
	Pomegranate (*Punica granatum*)	Leaves	[27]
	Coriander (*Coriandrum sativum*)	Leaves	[71]
	Spinach (*Spinacia oleracea*)	Leaves	[75]
	Blueberry (*Vaccinium corymbosum*)	Berry	[4,76]
	Grape (*Vitis vinifera*)	Berry	[4]
*A. arborescens*	Wheat (*Triticum aestivum*)	Grains	[77]
	*Celtis julianae*	Leaf	[21]
	Apple (*Malus domestica*)	Leaves	[78]
*A. infectoria*	Wheat (*Triticum aestivum*)	Grains	[22,79]
	Pyrethrum (*Tanacetum cinerariifolium*)	Leaves	[80]
*A. tenuissima*	Wheat (*Triticum* aestivum)	Grains	[79]
	Sugar beet (*Beta vulgaris*)	Leaves	[68]
	*Rhamnella franguloides*	Leaves	[81]
	Aloe vera (*Aloë vera*)	Leaves	[82]
	Celery (*Apium Graveolens*)	Leaves	[83]
	Amaranth (*Amaranthus hybridus*)	Leaves	[84]
	Tomato (*Solanum lycopersicum*)	Leaves	[36]
*A. triticina*	Wheat (*Triticum aestivum*)	Leaves	[85]
*A. poonensis*	Coriander (*Coriandrum sativum*)	Leaves	[71]
*A. alternariacida*	Potato (*Solanum tuberosum*)	Leaves, stems, tubers and fruits	[36]
	Tomato (*Solanum lycopersicum*)		[50]
*A. grandis*	Potato (*Solanum tuberosum*)	Leaves, stems, tubers and fruits	[86]
	Tomato (*Solanum lycopersicum*)		[23]
*A. dauci*	Carrot (*Daucus carota* subsp. *sativus*)	Leaves	[58]

## Data Availability

No new data were created or analyzed in this study. Data sharing is not applicable.
